# Supplementary material for: Content-rich biological network constructed by mining PubMed abstracts
Source: BMC Bioinformatics. 2004 Oct 8;5:147. doi: 10.1186/1471-2105-5-147 (PMC528731; doi:10.1186/1471-2105-5-147)
Supplement: Additional File 5 — The original Chilibot query results of the term "long-term potentiation (LTP)" and 22 other terms, limiting the latest references analyzed to the years 1990, 1995, 2000, and 2004. [file 1471-2105-5-147-S5.bz2 › chilibotAdditionalFile5/ltp2004/html/ARC.html]

 


**ARC** (Input: ARC ) 

---


**Google Searches:** Entire Web  | EDU domain only  | PDF files only

.

**External Links:** OMIM |
LocusLink
| Swissprot
| GeneCards

  
**Maps of ARC**

Simple
Complete
graph in
radiant
tree
square
layout.

**New Hypothesis !**

**Synonyms** 

- arc   [PubMed]

**Synopsis**

- Our results suggest that both NGFI A and **Arc** may regulate mechanisms of plasticity that had been invoked by heightened complexity of the visual environment.  Brain Res Mol Brain Res, 2002 
  [23]

- Our results are consistent with the hypothesis that developmental neurotoxicity of **ARC** is due, in part, to the presence of ortho substituted PCB congeners.  Toxicol Lett, 1998 
  [21]

- This induction requires the activity of PKA and mitogen activated protein kinase, suggesting a neuron specific pathway for the activation of arg3.1 **arc** expression.  J Neurosci, 2001 
  [19]

- Thus, **Arc** appears to play a fundamental role in the stabilization of activity dependent hippocampal plasticity.  J Neurosci, 2000 
  [19]

- We suggest that in developing embryos the neuralizing signal emanating from the organiser at first induces eIF4AII and the prospective neural crest in an **arc** low on the dorsal aspect of the embryo.  Development, 1997 
  [16]

- We will review findings from other laboratories that both LTP and memory are disrupted when **Arc** induction is blocked, and we will discuss the possibility that **Arc** protein may play a key role in activity dependent synaptic modification.  Neurobiol Learn Mem, 2002 
  [16]

- We have found that selective translation of **Arc** in a synaptoneurosomal preparation is induced by the brain derived neurotrophic factor, a neurotrophin that is released during high frequency stimulation patterns used to elicit LTP.  Proc Natl Acad Sci U S A, 2002 
  [16]

- All EGF induced responses were abolished by the specific tyrosine kinase inhibitor pp60v **arc** 137 157, suggesting that EGF receptor tyrosine kinase activity is essential for G i 1 2 mediated activation of PLC gamma 1.  Am J Physiol, 1997 
  [15]

- Moreover, the biotinylated derivative of **ARC** is able to deliver avidin, a non covalently attached protein cargo, into cells.  Bioorg Med Chem Lett, 2002 
  [14]

- These results demonstrate that ghrelin directly interacts with NPY neurons in the **ARC** to induce calcium signaling via PKA and N type calcium channel dependent mechanisms.  Diabetes, 2003 
  [14]

- The results support a model in which BDNF triggers long lasting synaptic strengthening through MEK ERK and selective induction of the dendritic mRNA species **Arc**.  J Neurosci, 2002 
  [14]

- The dynamics of trafficking of **Arc** mRNA reveal key features of the mechanism through which synaptic activity can both induce gene expression and target particular mRNA transcripts to the active synapses.  Proc Natl Acad Sci U S A, 2001 
  [11]

- At the age when these animals developed cognitive dysfunction, they had reduced mRNA expression of several genes essential for long term potentiation  [LTP]  and memory formation **Arc**, Zif268, NR2B, GluR1, Homer 1a, Nur77 TR3 .  J Neurosci, 2003 
  [11]

- Local infusion of the transcription inhibitor actinomycin D ACD 1 hr before or immediately before BDNF infusion inhibited BDNF LTP and upregulation of **Arc** protein expression.  J Neurosci, 2002 
  [10]

- Arcand H1a may function in concert in the structural and functional modifications of dendrites that lead to long term changes in synaptic efficacy.  J Neurosci, 2002 
  [10]
